# cellSTORM—Cost-effective super-resolution on a cellphone using dSTORM

**DOI:** 10.1371/journal.pone.0209827

**Published:** 2019-01-09

**Authors:** Benedict Diederich, Patrick Then, Alexander Jügler, Ronny Förster, Rainer Heintzmann

**Affiliations:** 1 Leibniz Institute of Photonic Technology, Albert-Einstein-Straße 9, Jena, Germany; 2 Institute of Physical Chemistry and Abbe Center of Photonics, Friedrich-Schiller-University, Jena, Germany; University of Manchester, UNITED KINGDOM

## Abstract

High optical resolution in microscopy usually goes along with costly hardware components, such as lenses, mechanical setups and cameras. Several studies proved that Single Molecular Localization Microscopy can be made affordable, relying on off-the-shelf optical components and industry grade CMOS cameras. Recent technological advantages have yielded consumer-grade camera devices with surprisingly good performance. The camera sensors of smartphones have benefited of this development. Combined with computing power smartphones provide a fantastic opportunity for “imaging on a budget”. Here we show that a consumer cellphone is capable of optical super-resolution imaging by (direct) Stochastic Optical Reconstruction Microscopy (*d*STORM), achieving optical resolution better than 80 nm. In addition to the use of standard reconstruction algorithms, we used a trained image-to-image generative adversarial network (GAN) to reconstruct video sequences under conditions where traditional algorithms provide sub-optimal localization performance directly on the smartphone. We believe that “*cell*STORM” paves the way to make super-resolution microscopy not only affordable but available due to the ubiquity of cellphone cameras.

## 1 Introduction

Super-resolution by Single Molecule Localization Microscopy (SMLM) techniques like Photo-Activated Localization Microscopy (PALM) [1, 2] or (direct) Stochastic Optical Reconstruction Microscopy (*d*STORM) [3] is well established in biology and medical research. Together with other modalities like Stimulated Emission Depletion (STED) [4, 5] and Structured Illumination Microscopy (SIM) [6, 7], SMLM revolutionized optical far-field microscopy beyond the Abbe limit [8].

Typically these methods rely on costly hardware for excitation and detection [9, 10]. Scientific-grade sCMOS or emCCD cameras are a major cost factor, as high photon efficiency and low noise are paramount. However, previous studies [10, 11, 12] have successfully demonstrated that substituting major elements from a SMLM setup, such as the sCMOS camera by a low-cost CMOS camera, or microscope stands by off-the-shelf or even 3D-printed parts, stil yields super-resolution. The evolution of mobile phones created surprisingly powerful cameras and sensors backed by respectable computational power worth considering as an alternative.

This, combined with their widespread availability gave rise to the developing field of mobile microscopy, which so far resulted in hand-held devices capable of quantitative phase-imaging of biological material by combining the cellphone with customized hardware adapters [13, 14] or act as portable diagnosis devices to detect e.g. waterborne parasites [14, 15, 16]. In regions, where proper working conditions for ordinary lab equipment are hard to establish (e.g. due to high humidity) cellphones might represent a good alternative to expensive and potentially fragile cameras. Furthermore the densly integrated functions of a cellphone allows acquisition and processing on the same device, making parallel imaging scenarios very attractive.

Most smartphone camera sensors are equipped with Bayer patterned filters, significantly lowering the detection efficiency compared to monochromatic imaging. Some recent cameras modules, such as in the Huawei P9, feature sensitive monochromatic CMOS camera chips. However, acquiring high-quality RAW data using a cellphone is nevertheless very challenging. Hardware abstraction layers embedded into the firmware of the camera module prevent accessing the raw pixel values. Compression and noise artifacts are therefore a potential problem of imaging with smartphone cameras.

We show that by simple adaption of the mobile phone device to a common widefield microscope equipped with an excitation laser it is possible to image well below the diffraction limit. Additionally we present a novel machine-learning-based image processing algorithms being able to process *d*STORM experiments directly on the device. This allows making existing setups even more portable and affordable.

## 2 Results

### 2.1 Cellphone data acquisition

In order to image the blinking fluorophores, we attached the smartphone (P9 EVA-L09, Huawei, China, Table 1) directly to the eyepiece of a standard inverted research microscope (AxioVert 135 TV, Zeiss, Germany) with a 3D-printed interface. For all experiments we used a 12 Bit monochromatic sensor chip (Sony IMX 286, Japan, Table 1, [17]) of the P9’s dual-camera module. The aim of the camera manufacturer is to ensure optimal image quality in everyday environments. Tailored algorithms help to hide problems introduced e.g. by the small pixels and lens dimensions [18, 19].

**Table 1:** Comparison of a scientific-grade with a low-cost cell phone camera.

	Andor iXonEM+ 897	Huawei P9 (EVA-L09, Sony IMX286, Grayscale
Pixel#:	512 × 512	3980 × 2460
Sensortype	(back-illuminated) emCCD	(back-illuminated) CMOS
Pixelsize (*μ*m):	16	1,25
Bitdepth:	14 Bit	12 Bit
Read Noise (*e*^−^):	0,2 RMS	1,23 RMS (see Methods 5.3)
Quantum-Efficiency	≥ 90%	≈ 70 − 80%
Price	≈ 20 *k*$	≈ 300 $ (camera module: ≈ 20 $)

In contrast to industry-grade CMOS cameras, the acquired images of cellphone cameras are post-processed by proprietary firmware, called the image signal processor (ISP) [20, 21]. This allows real-time optimization of the image quality. It is not only responsible for demosaicing the Bayer-pattern to generate RGB images, but also reduces the effect of lens aberrations and removes hot-pixels or thermal noise [18, 22]. Additionally it provides hardware control (e.g. autofocus, optical image stabilization) and encodes the video-stream into less memory-consuming formats.

Modern cellphones offer the raw camera sensor pixel values (“*snap-mode*”), i.e. the sensor data before further processing or compression by e.g. JPEG/MPEG algorithms [15, 23, 24, 25]. However, *d*STORM requires continuous and fast acquisition over several minutes to record sufficient fluorescence events for image reconstruction, which is incompatible with snap-mode acquisition. The computational effort to save a time-series of raw-frames makes it impracticable for these measurements. Hence, we were forced to use the standard time-series acquisition mode (“*video-mode*”), where the compression of the raw data was unavoidable.

Acquiring monochromatic video-sequences is not part of the cellphone’s software. We thus wrote a customized application (APP) based on the open-sourced camera library “FreeDcam” [26, 27], which enables the full control over the camera parameter like sensitivity (ISO), focus position, exposure time and frame-rate, as well as the access to the monochromatic chip. The “FreeDcam”-based APP works on any device, but takes only full advantage of devices equipped with monochromatic sensors.

Down-converting the video-stream, e.g. using the H264 video-codec, is also implemented on the ISP. To reduce the amount of memory, it relies on the exact-match integer transform [28] which uses reference images and calculates residual/difference images to reduce the amount of redundant information. This lossy compression partly obscures accurate information of the pixel, necessary for precise localization of the fluorophores.

### 2.2 Localization based on machine learning algorithms

In general, machine learning (ML) has the ability to create an implicit model which maps a set of input variables onto a set of outputs [29, 30, 31, 32]. A large variety of different network architectures applied to image processing problems have shown that using prior knowledge, single-image super-sampling [33] or recovering the optical phase from an intensity image [34] is possible.

Motivated by recent approaches where an adversarial network architectures (GAN) [35] was trained on a noise model [32] or the variational auto-encoder-based neural network (VAE) which directly localize STORM events [31], we propose a localization algorithm which accounts for strongly compressed noisy data (see Methods 5.3). Compared to the VAE, the GAN architecture of the neural network (NN) has the ability to be used more generically. We found that, once trained, it serves as a parameter-free localization method for multi-emitter fitting in compressed image-streams as well as image sequences coming from an emCCD camera chip with different SNR ratios.

**Fig 1:**
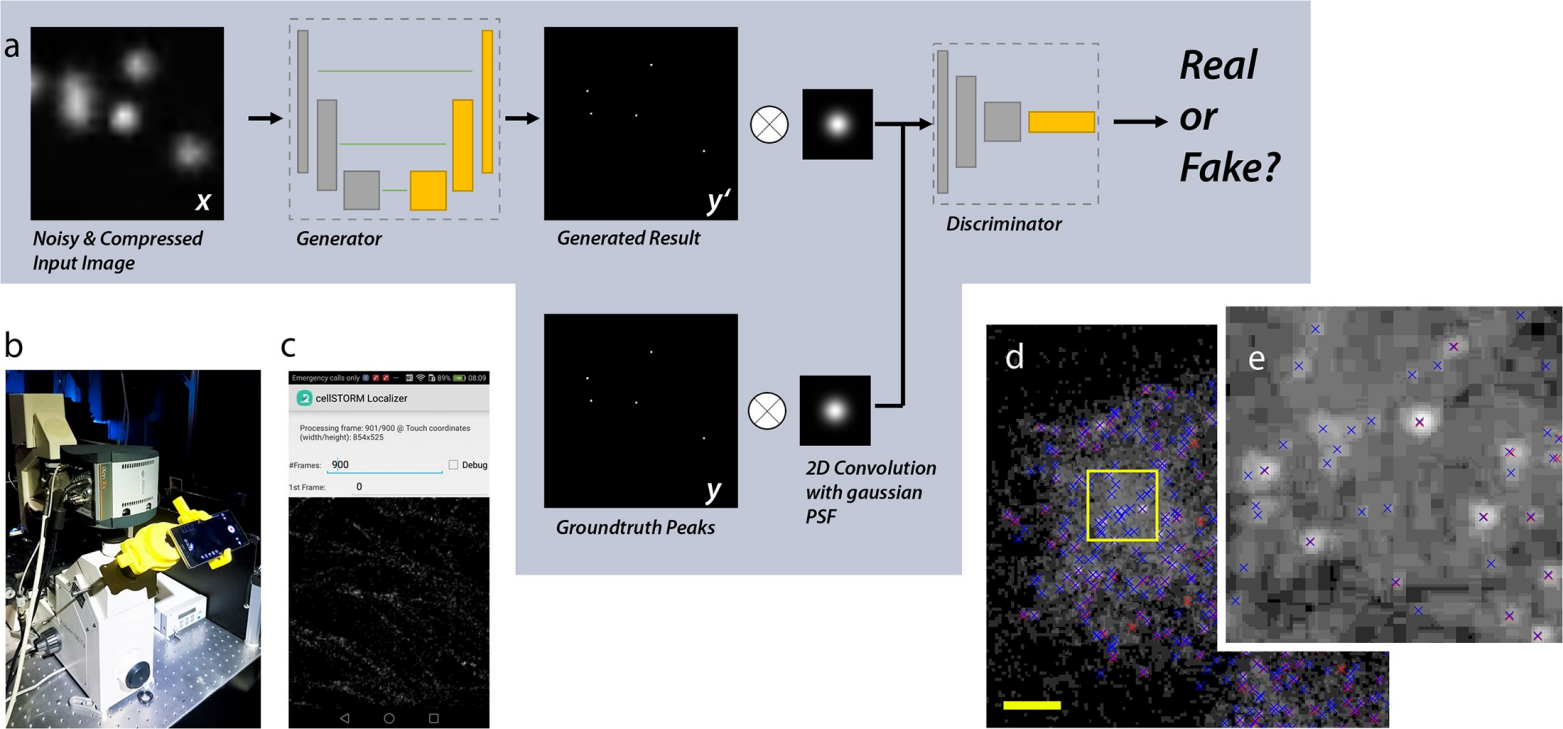
Architecture and results from the GAN. (a) The image-to-image GAN architecture tries to find a generator-model which maps compressed and noisy input images to a localized version of the PSF’s origin. Image (d zoomed in e) shows a sample-frame (log-scale) from real *d*STORM measurements localized with ThunderSTORM (blue) and the NN (red). The number of falsely detected emitters is significantly lower in data processed by the NN. (b) shows the custom made, 3D-printed cellphone adapter attached to the Zeiss Axiovert microscope body holding the HUAWEI P9 running the cellSTORM Localizer APP shown in (c).

Following a modified version of the popular image-to-image GAN (Pix2Pix) [36] network architecture (see Methods 5.4 and Fig 1a the goal of our network is to detect the origin (e.g. center position) as a pixel on a supersampled grid of a blinking fluorophore in a degraded video-frame of *cell*STORM measurements (“*x*”). The sum of all these individual reconstructed frames yields the final reconstruction (“*Y*”).

Compared to conventional approaches, based on encoder-decoder networks [37], the cost-function for the training process is not defined a priori but trained alongside the generator. This learned cost-function often fits the model better and avoids unwanted blurring of the results as often caused by using the L2-norm during training [36, 38].

Standard localization software like ThunderSTORM [39] applied to H.264-compressed noisy data showed many false localizations (indicated as blue markers in Fig 1d and 1e) whereas the trained NN successfully filtered them (indicated as red markers in Fig 1d and 1e). The network’s output is a table with the pixel-coordinates of local maxima of all localized events and a summation of all processed frames to produce a super-resolved image.

To ensure fast convergence of the network, we added prior information to the loss-function, which emphasize sparsity of the localized events. The adversarial loss was further able to localize emitters where traditional algorithms showed suboptimal localization performance.

Our training datasets relies on mixed simulated as well as experimental data (further details in Methods 5.4.4 and 5.4.4). We create a ground truth (GT) stack of randomly blinking events with known positions *y* in ThunderSTORM, where noise based on our camera model (Methods 5.3) and video-compression was added to simulate realistic camera frames *x*. Experimental data was produced by localizing an image-sequence of *cell*STORM measurements with ThunderSTORM and produce image-pairs with the detected origin of the molecules rendered as single pixel events *y* and their corresponding measured frame *x*. Mixing the datasets enables to learn a generic representation of the data and allowed to outperform ThunderSTORM’s [39] price-winning localization algorithm [40] in terms of localization-accuracy at low light conditions (Sec. 2.4).

For all results shown here we used the same trained NN. Note, that the model can easily adapt to different camera characteristics (e.g. sensor, compression, SNR) and experimental conditions (e.g. fluorophore, etc.) depending on the generated training samples.

#### 2.2.1 Localization on the cellphone based on machine learning

Finally, since Tensorflow [41] allows exporting and executing the trained networks to e.g. cellphones, we wrote a custom APP [42] based on Tensorflow and OpenCV [43] which directly localizes a recorded video-stream on the device. This makes additional computational hardware redundant and promotes the cost-effective realization of super-resolution measurements to a greater extent. So far we were able to achieve up to 9 fps while localizing a video-stream with 64×64 pixels, serving as a proof of principle. A further description of the method can be found in Sec. 5.4.6.

### 2.3 Localization-results of compressed smartphone data using standard and machine learning algorithms

The reduced signal-to-noise ratio (SNR) and blocking artifacts from video compression are apparent in the image sequences acquired by a cellphone camera (Fig 1d). Nevertheless, the robust nature of the reconstruction algorithms in rapidSTORM [44] and ThunderSTORM [39] successfully localized blinking events even under non-ideal conditions. Both algorithms yielded comparable results when reconstructing the final image from recorded data. The output was also used to verify our NN’s result to demonstrate the correct functionality.

**Fig 2:**
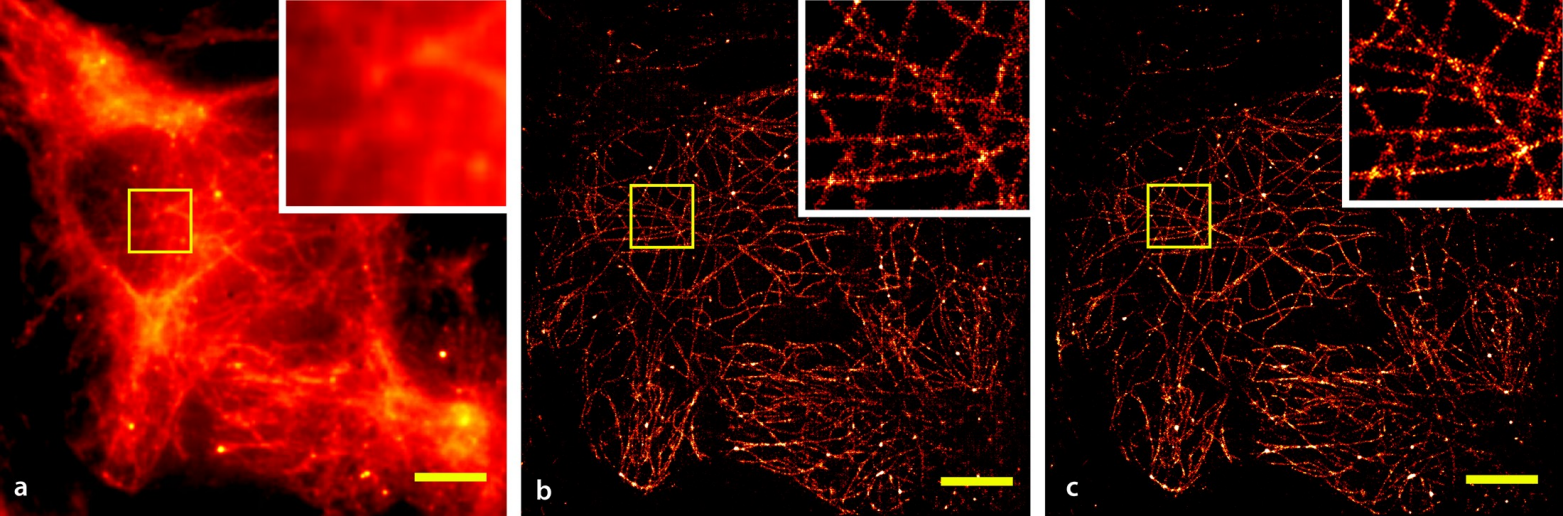
Comparison of cellSTORM results using NN and ThunderSTORM. Microtubuli in HeLa cells stained using AlexaFluor 647 labeled antibodies and recorded with the cellphone camera. (a) A widefield-equivalent image obtained by summing over all recorded frames, (b) the reconstructed result based on summing the NN-processed frames and (c) the output of the localization software ThunderSTORM using optimal parameters (no drift correction). (Scalebar = 3 *μm*, 3×Zoom in the yellow-boxed ROI).

Fig 2 shows the results obtained with *cell*STORM from HeLa cells stained for tubulin using AlexaFluor 647-labeled primary/secondary antibodies. Applying both methods, NN-based localization (Fig 2d) and ThunderSTORM (Fig 2e) directly to the approximately six thousand acquired video frames (at 20 fps), resolve the structure of microtubuli at a resolution of 75 nm measured by the Fourier ring correlation (FRC) [45]. To compare to conventional *d*STORM data, we recorded another series of a similar cell using the emCCD camera of our setup. Due to the low photon yield at the cellphone camera, we opted against using a beamsplitter to simultaneously record the same area with the cellphone and the emCCD camera and instead imaged separate cells of the same sample.

The images acquired using the professional emCCD camera under identical buffer and illumination conditions (see Methods 5.2) yielded a final resolution of 45 nm. While this number is smaller than for *cell*STORM the relative difference is nonetheless surprisingly small.

**Fig 3:**
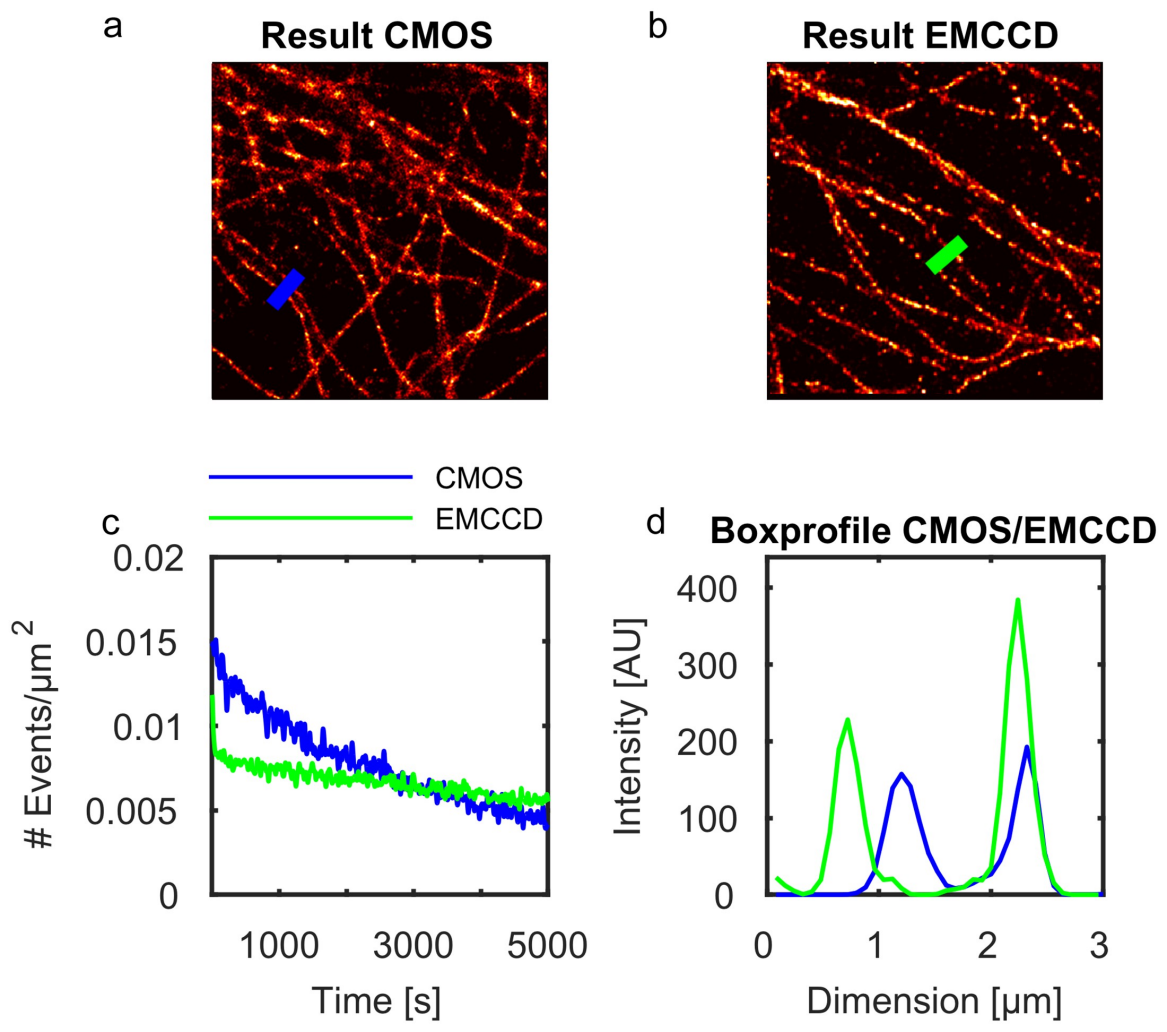
Comparison over detected events per area-unit/box-profile. a) Shows the number of detected events per unit-area in each frame in case of the CMOS (blue) and EMCCD (green) camera. Fig b) and c) show reconstructed results from *d*STORM measurements, where we plotted the box profile (6 pixels) along the lines in each image in figure d).

A further analysis of the resolution was derived from several box-profiles (6 pixel edge length), where one is plotted exemplary in Fig 3d. The localization was done with ThunderSTORM, to have equal parameters for the analysis. It can be seen, that the full with half maximum (FWHM) in the cellSTORM measurements is slightly lower compared to the one coming from the emCCD which goes along with the FRC measurements.

Additionally we tried to get an impression of how many events per area-unit will be detected by the emCCD compared to the CMOS camera. To this aim we counted the events over an FOV of about 100 × 100 *μm*^2^ in each frame. The blinking statistics is highly varying from sample to sample. Therefore we have chosen a FOV, where the measurements of the emCCD and CMOS produced similar results. In case of the CMOS camera, ThunderSTORM detects slightly more events. This could be due to the compression artifact which we further analyze in Sec. 2.4.

### 2.4 Analysis of the NN-based localization approach

Especially under poor imaging conditions, applying our learned black-box model, described in section 2.2, turned out to be beneficial. In several attempts to reconstruct the recorded video-stream using ThunderSTORM, we observed a grid-like pattern in the localized result (Fig 5a) which is likely due to the 4×4 block exact-match integer-transform of the H.264 codec. It introduces abrupt changes in the local intensity, causing the localization algorithms to wrongly identify events. Particularly at low-light conditions, the SNR decreased dramatically, further emphasizing this effect (Fig 1d/1e, blue markers). In contrast, our NN-based approach reduced such artifacts significantly by filtering false-positive events Fig 1d/1e, red markers) and the final result in Fig 5b.

**Fig 4:**
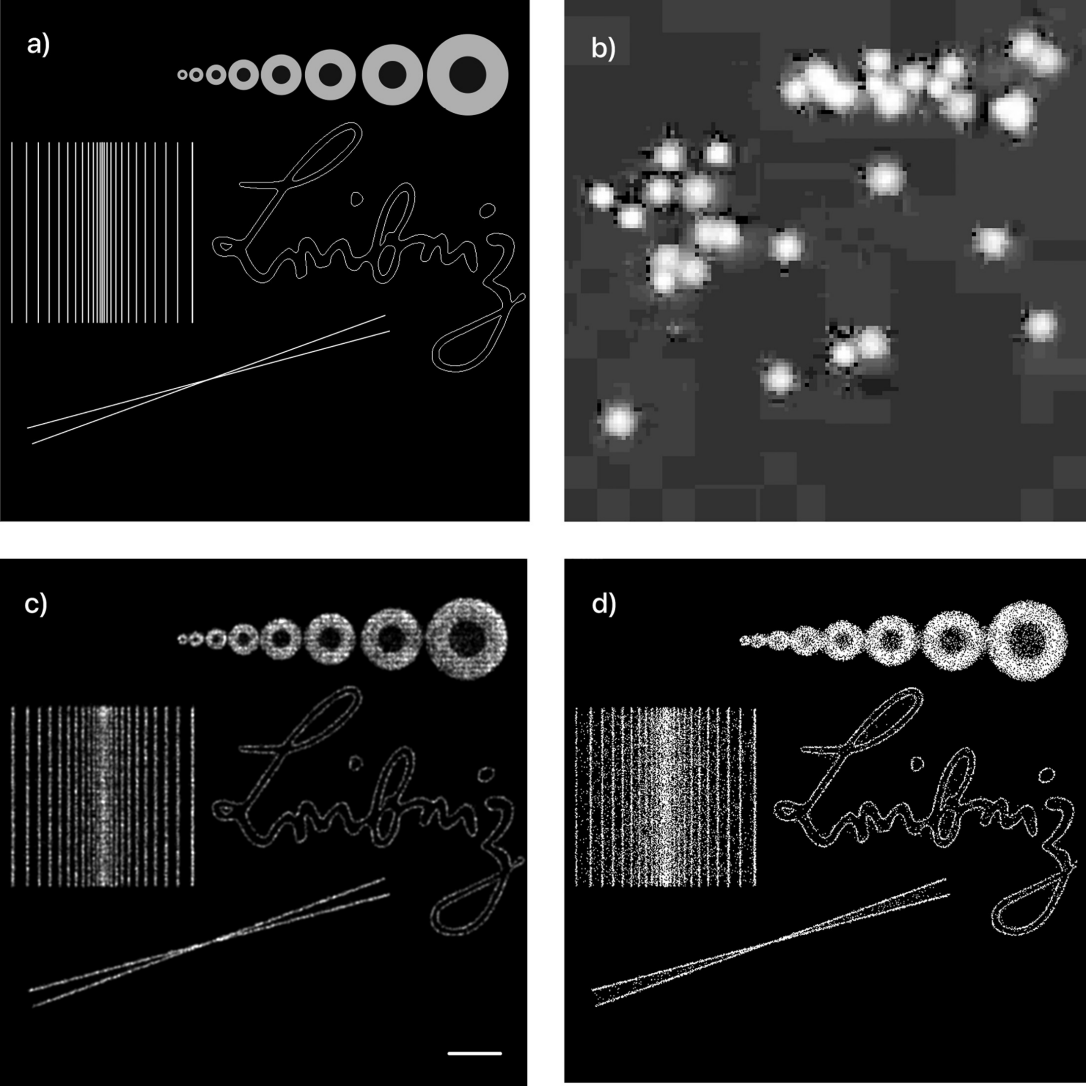
Qualitative analysis of cellSTORM. STORM-datasets created with ThunderSTORM. a) Ground truth of created test-target which is the basis for the generated STORM datasets with different H.264-compression rates and number of photons. b) Individual frame of the generated STORM-datastack with compression artefacts (1000 photons/fluorophores; compression ratio: 70%; resized, nearest-neighbors: 6×; log-scale), Reconstructed image with our NN c) and ThunderSTORM d) (Scale bar: 1 *μm*).

To afford a quantitative comparison of how well the NN and ThunderSTORM recover data suffering from noise and compression artifacts, we measured the mean euclidean distance between all detected events and their corresponding ground truth events for every frame. An artificial STORM dataset of the Leibniz-IPHT institute’s logo and other test structures was generated in ThunderSTORM (Fig 4a). The stack (2000 frames, emitter density of 6/*μm*^2^, Fig 4b)) was processed by our camera model (Methods 5.3) with varying compression quality (70%, 80%, 90%, 100%) to simulate different compressions that may occur in other smartphones. The number of photons per emitter (50, 100, 500, 1000) is also varied to demonstrate the functionality even below the normal achievable range of common fluorophores in Alexa647 (≥ 500 detected photons/fluorphore [46]). We processed all frames in ThunderSTORM using the same set of parameters (i.e. optimal result also for low SNR) to mimic the parameter-free localization procedure compared to the NN.

**Fig 5:**
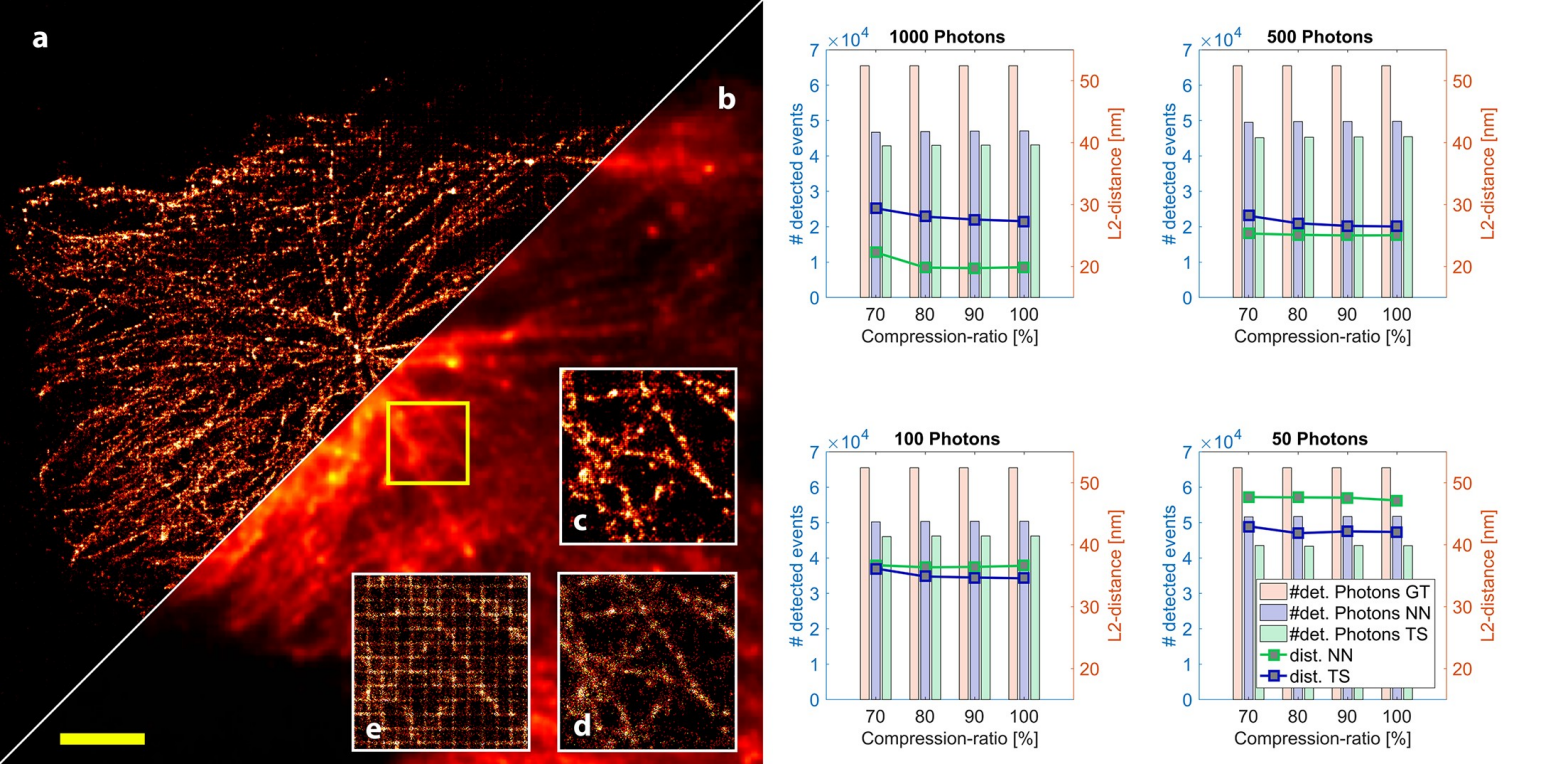
Quantitative and qualitative analysis of cellSTORM. Results after summing all frames processed by the NN a) and directly coming from the camera b). When processing the video-sequence in ThunderSTORM, it introduces a checkerboard-like pattern shown in the two-fold zoomed version of the yellow box in e), which can be reduced by adjusting the peak intensity threshold (e.g. 3 ⋅ *std*(*frame*)) illustrated in d). c) shows the NN’s result successfully compensating for the pattern effect, due to high noise and compression of the video stream. Scalebar = 5 *μm*. The graphs on the right hand side visualize a comparison of the achieved localization accuracies of our NN and ThunderSTORM applied to simulated data. We varied the number of photons per emitter (1000, 500, 100, 50) as well as the compression ratio of the H.264 codec (70-100%), before the video-stream was localized by the NN and ThunderSTORM. We estimated the accuracy by measuring the Euclidean distance between a nearest neighbor in a GT and reconstructed frame and calculate the mean over all distances, visualized as green (NN) and blue (ThunderSTORM) plots. The green (NN) and blue (ThunderSTORM) bars indicate the number of correctly detected emitters within the allowed range of 200 nm compared to the 65.489 GT events (orange). It can be seen, that the NN always detects more good quality emitters, but with a loss of accuracy at lower intensities (i.e. $\leq 500\;\frac{photons}{emitter}$) compared to ThunderSTORM.

From the results in Fig 5 it is clearly visible that the NN outperformed ThunderSTORM in terms of number of correctly detected events (only considering GT-neighbors closer than 200 nm) in all measurements. It also yielded better accuracy in situations with more photons per emitter.

Nevertheless the accuracy degrades in low-light situations ($\leq 500\;\frac{photons}{emitter}$) where ThunderSTORM achieves better localization accuracies at a price of less localized events. Here the NN exhibited around 20% more correctly detected events (blue bars in Fig 4) at the expense of slightly reduced accuracy. This effect worsened at higher compression ratios.

Although the NN was not explicitly trained on a specific SNR-/compression-ratio, the improved results in the reconstruction above 500 $\frac{photons}{emitter}$ can be due to the composition of the training data. Besides simulated data it also contains experimental measurements from samples stained with Alexa647, typically emitting $\geq 500\;\frac{photons}{emitter}$. This potentially indicates better results in this area and suggests that the NN can behave even better with tailored training-datasets in terms of expected photon statistics. However this goes along with reduced generality.

## 3 Discussion

We have demonstrated the suitability of using a modern smartphone camera for imaging beyond the diffraction limit. So far, unavoidable limitations imposed by current smartphone hard- and software, i.e. low-light performance and artifacts caused by compression and image “enhancement” algorithms, prevented the use for high-quality imaging. Nevertheless we have been able to resolve sub-diffraction detail in cytoskeletal structures on a level similar to conventional *d*STORM setups.

The nearest neighbor analysis of the ground truth data of a simulated STORM data-stack localized with ThunderSTORM and the NN demonstrates the strength of our NN approach. Out-of-focus or artifactual localization events were successfully suppressed. Especially for low-photon statistics as in the *cell*STORM experiments, the NN approach clearly outperformed ThunderSTORM in terms of detected localizations and average localization accuracies (calculated as the mean deviation from the ground truth). This means our NN has the potential to perform even better in explicitly trained (real world) conditions and to easily customize the localization for each experiment individually.

The trained NN proved to be a robust and generic way to reconstruct *d*STORM data acquired by a smartphone sensor at poor imaging conditions, with sometimes a small loss of localization uncertainty. Additionally it serves as a blue-print for rapid software prototyping on mobile devices as the steps of computational expensive (pre-)processing as well as exhaustive debugging are carried out on desktop machines. The result can then easily be implemented on e.g. modern cellphones enabling e.g. diagnostics or telemedicine in the field.

## 4 Conclusions

We showed that widely available cellphone cameras can be used for SMLM, yielding image quality approaching the performance of much more expensive professional cameras. This is an important contribution to the development of an overall cost-effective and potential portable SMLM system. In the future our work can be combined with with on-chip localization techniques like [47] and other parts like lasers and objective lenses can be substituted with inexpensive components.

It should also be noted, that the aspect of transferring camera characteristics (e.g. train on emCCD data and deploy it on cellphone cameras) should be further investigated, as we see potential to further improve the localization accuracy.

New cellphones with dedicated NPUs give hope to further accelerate the convolutional processing of the data, which makes on-device reconstruction more attractive. This not only advances the system’s simplicity and usability, but dramatically lowers the costs.

This makes cutting-edge scientific instruments not only affordable but also available to involve an even larger community. Educational environments, where ordinary cellphones are readily available, directly benefit from our approach. This removes barriers for future research of all levels of society and could bring new contributions to the field of biological and medical research.

## 5 Methods

### 5.1 Optical setup

The basic *d*STORM-system is realized with a standard inverted microscope stand (AxioVert 135 TV, Zeiss, Germany) equipped with a nosepiece-stage (IX2-NPS, Olympus, Japan) to keep drift low. A 637 nm diode laser (P = 150 mW, OBIS, Coherent, USA) is focused to the back-focal plane of the microscope objective lens (ZEISS 100×, NA = 1.46) to realize a homogenous illumination in the sample plane. Using an adjustable mirror, it is also possible to change the laser position in the back-focal plane. This enables background reduced total internal reflection (TIRF) illumination. The microscope can potentially be replaced by a low-price customized optical setup also relying on cheap lasers [12], making it available for about ≪ 10*k*$.

**Fig 6:**
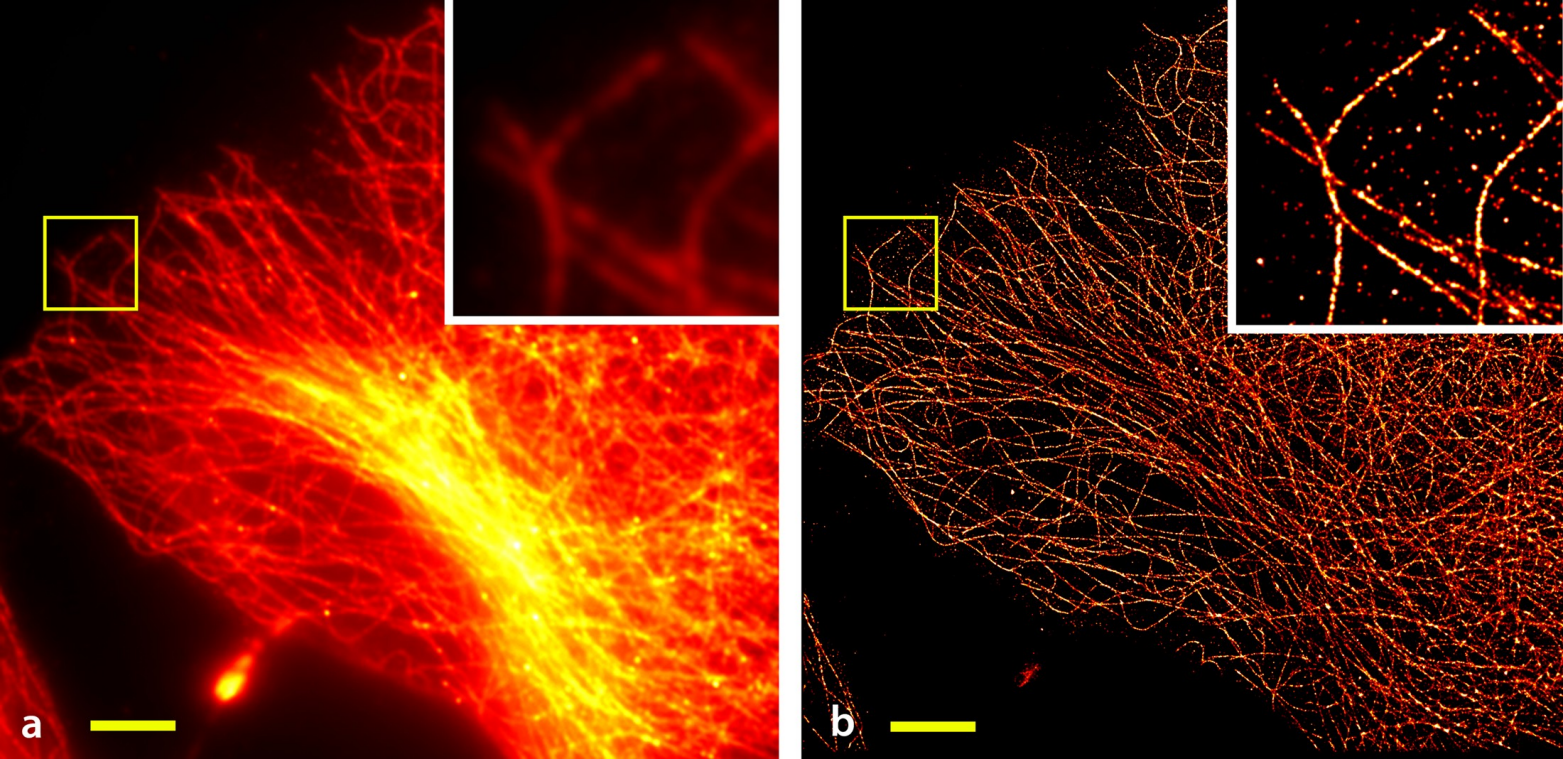
Result from the emCCD camera. A widefield equivalent image obtained by summing over all recorded images from microtubuli in HeLa cells stained using AlexaFluor 647 labeled antibodies recorded with the Andor emCCD camera (a) and the image after reconstruction with rapidSTORM (b). No drift correction was applied to the time-series. (Scalebar = 6 *μm*, 3×Zoom in the yellow-boxed ROI).

An emCCD camera (iXon3 DU-897, Andor, UK, Table 1) can be used to image the sample in widefield and STORM-mode during normal operation (*d*STORM reconstruction result Fig 6). For imaging via the cellphone, the beam-path is switched from the camera port to the eyepiece, where a common 10× monocular eyepiece is equipped with a custom-made 3D-printed cellphone adapter [48]. The cellphone (P9 EVA-L09, Huawei, China, Table 1) is placed with its camera lens in the Ramsden disk of the eyepiece (see Fig 1b), since an eyepiece images the intermediate image produced by the tube lens of the Axiovert body to infinity.

### 5.2 dSTORM imaging samples and results from an emCCD camera

HeLa cell samples have been prepared using the PFA-fixation protocol outlined in [49]. Microtubuli have been stained using monoclonal mouse anti-*β* -tubulin (Sigma Aldrich) and goat anti-mouse IgG secondary antibody (ThermoFisher Scientific), labeled with Alexa Fluor 647 at 1:150 and 1:300 dilution, respectively. All imaging experiments have been conducted in imaging buffer prepared freshly from 150-200mM MEA (*β*-Mercapto-ethylamine hydrochloride) in PBS and pH adjusted to 7.4 using NaOH. The oxygen scavenging effect from MEA has been proven efficient enough to refrain from additional enzyme-based oxygen scavenger systems.

### 5.3 Camera characterisitics

**Fig 7:**
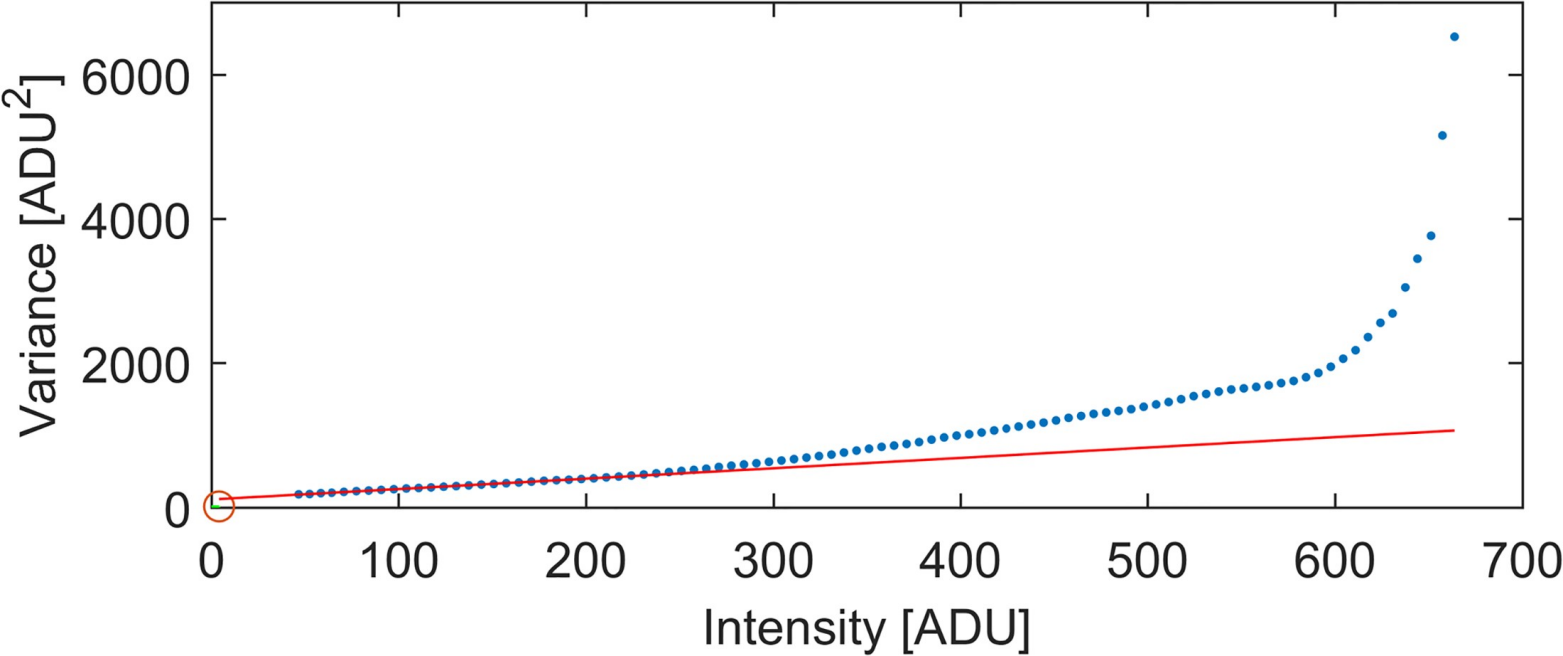
Mean-variance plot HUAWEI P9. Mean-variance plot generated using a series (ISO = 3200) of unprocessed raw images (”snap-mode”) acquired by a Huawei P9 camera (blue points). The camera gain is constant up to an critical intensity of 220 ADU, which should not be exceeded in the experiment.

A characteristic mean-variance plot is generated for the Huawei P9 camera, from a set of 10 unprocessed raw images (snap-mode) acquired in 12Bit (DNG) of an intentionally defocussed but stationary object (see Fig 7) by using the Dip-Image [50] “cal_readnoise” routine.

It can be seen that the variance does not increase linearly with the mean intensity as it should for a shot-noise-limited sensor [51]. The slope of the curve is the gain, which is constant up to an intensity of around 220 ADU. The noise parameters extracted from the linear low-intensity range of the curve are: offset = 4,1 ADU; gain = 0,69 *e*/ADU; readnoise(Bg) = 2,5*e*^−^RMS; at an ISO3200, which was also used during our measurements. In order to have a linear gain, the camera should not be exposed to much, so that the pixel values do not exceed the critical intensity value of 220 ADU.

Especially noteworthy is the low readnoise. However, it cannot be guaranteed that the hardware-based preprocessing especially in the video-mode does not alter this value.

**Fig 8:**
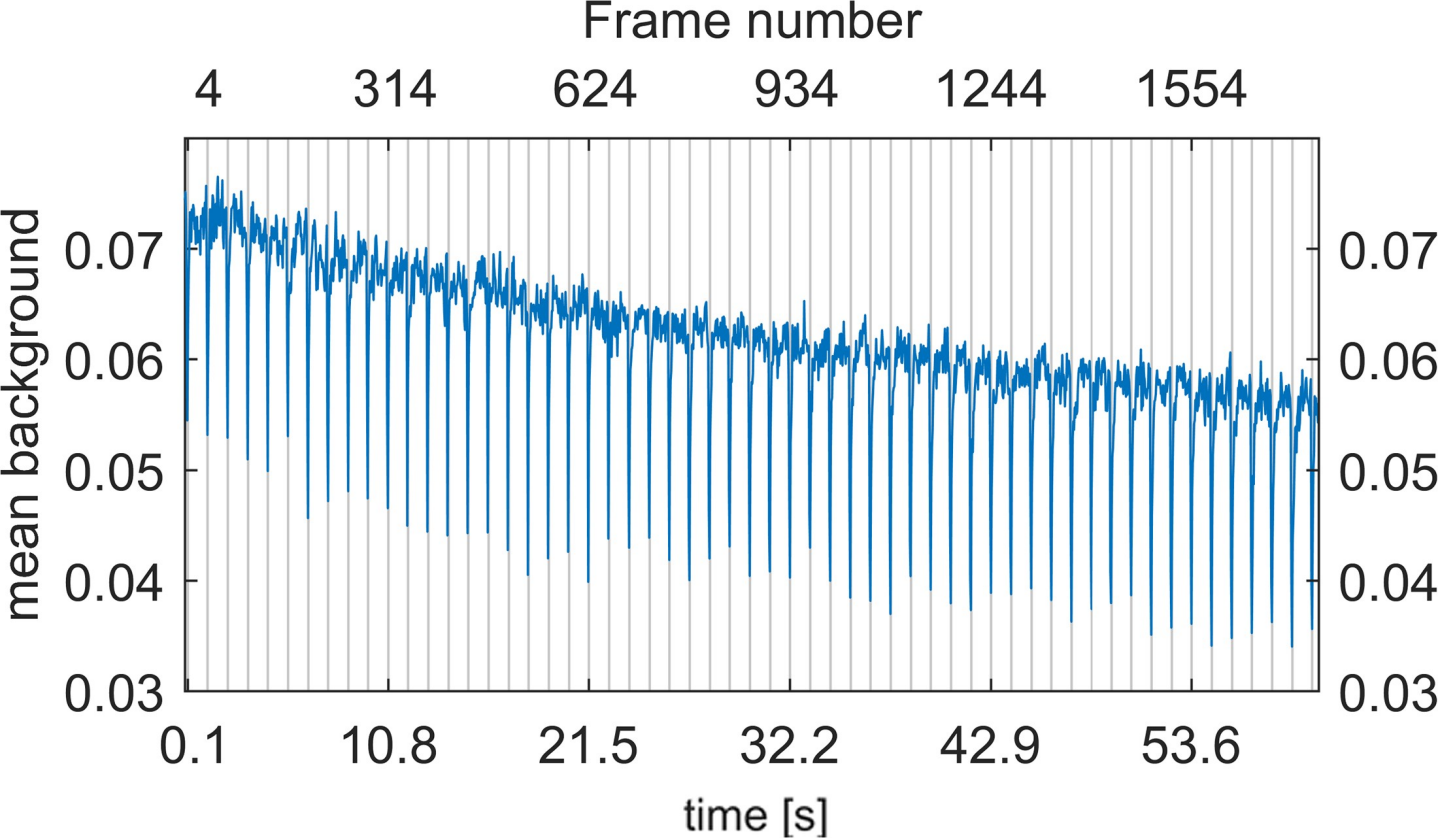
Mean of a dark image over time. Mean of a dark image acquired with a Huawei P9 camera and compressed with a H.264 encoder as a function over time. The overall decay of the signal and the periodic drop of the signal demonstrates the disadvantages of a compressed video signal. Equidistant gray lines (vertical) with 1.07 s (i.e. each 31st frame) at 29 *fps* spacing show the periodicity of the drop in the intensity signal. Certain pixel values less than a threshold are clipped to zero.

A series of ≈ 8000 images of dark background acquired in the video-mode with the Huawei P9, automatically compressed with a H264 encoder, shows a problematic property. The mean of each dark frame over time (subset of 60*s*) is shown in Fig 8. It can be seen that the overall background drifts over time. This might be a thermal problem, although the signal is expected to rise rather than drop. This effect, however, might also be caused by the compression of the incoming signal. Unfortunately the Huawei P9 has neither a temperature sensor on the chip nor a reproducible data compression, so the cause remains unclear. In addition, the signal drops periodically (every 1.07 s at 20 fps) which seems to be a compression artifact. A homogeneous although slightly noisy line would have been expected. The video acquisition exposes the drawback and limitations of the compression.

Looking at Fig 8 it can be seen that both the P9’s sensor and the video codec influence the saved image in a periodical but unpredictable manner. Settings like framerate and bitrate of the video-codec alter this effect. In addition, we observed that the dips depend on the number of photons. Thus, it is difficult to correct the compression-related artifacts of an unknown image.

Imaging techniques that require extensive image processing will have problems using such data. However, wide-field microscopy of bright specimens will be less affected. The localization accuracy of SMLM will be impaired the more noise each raw image contains.

Yet noise allone will not create artifacts. The occurring drop of the overall intensity is no problem either, because each image is processed individually and offset variations are automatically accounted for by rapidSTORM, ThunderSTORM as well as our cellSTORM.

### 5.4 Neuronal network architecture and training

In the beginning we used NNs to enhance the recorded image sequences which where then fed into common localization software like rapidSTORM [44] and ThunderSTORM [39]. This has the advantage to benefit from the already existing and robust localization algorithms. Unfortunately this led to only minor improvements and in many cases actually to a deterioration of the reconstructed results, therefore we followed the approach from Nehme et al. [31] to directly generate localization maps from the blinking fluorophores.

#### 5.4.1 Architecture

The network receives decoded and upsampled (e.g. 5×) video-frames *x* and their corresponding ground truth localization maps *y*.

The data-pairs (see Section 5.4.4) were fed into our modified version of the image-to-image GAN network [36], implemented in the open-source ML library Tensorflow [41]. The code is based on the open-sourced version described in [52] and is publicly available [53].

To circumvent a checkerboard-like artifact resulting from the generator in the reconstruction process, we replaced the transposed-convolution operation in the decoding step of the U-NET [30] by a resize-convolution layer as suggested in [54]. This in combination with longer training procedure eliminates high-frequency patterns due to the low coverage of the convolutions in the deconvolution process of the U-NET.

The floating-point localization table is generated by converting pixel-values greater than 0.3 ⋅ *max*(*I*_*frame*_) into effective pixel-dimensions.

#### 5.4.2 Training

The neural network (NN) was trained on a Nvidia Titan X GPU over fifteen thousand samples (input-size 256×256) with equal acquisition parameters corresponding to the *d*STORM experiment based on methods presented in Methods 5.4.4 and 5.4.4. Data was mixed in equal parts (i.e. 50%/50%) to not only learn the model of ThunderSTORM. We use minibatch stochastic gradient descent (SGD) and relied on the ADAM optimization scheme [55] with learning rate of 1 ⋅ 10^−4^ and momentum of beta = 0.25.

Our experiment showed, that the training converged to equilibrium after 10 epochs at a batch-size of 4 frames, which took about 3h time-effort on an ordinary desktop machine with 64Gb RAM, Intel Xeon octacore and a Nvidia TitanX graphics card with 12GB memory. It is worth noting, that a precise alignment of the data is crucial, otherwise the recovered events will be shifted by an unknown amount and the localization fails due to smeared-out blinking events.

#### 5.4.3 Cost-function

Following the original Pix2Pix-approach in [36], the conditional GAN-loss can be expressed as

$$L_{cGAN}\left(G,D\right)=E_{x,y}[log(D(x,y))]+E_{x}[1-log(1-D(x,G(x))]$$ (1)

where *x* gives the degraded video-frame and *y* the input ground truth images. *G* corresponds to the U-NET generator which tries to map the input image *x* together with random noise *z* to the recovered output-frame *x*; *G*: {*x*} → *y*. The discriminator *D* has to distinguish between real or fake (e.g. produced by the generator) samples. Additionally the $L_{1}$-norm is given as

$$\begin{array}{c} L_{1}\left(G\right)=E_{x,y}[{∥y-G\left(x\right)∥}_{1}]. \end{array}$$ (2)

For stable training with faster convergence, we convolved the fluorophore location maps (indicated as intensity peaks) in input *y*, as well as the generated predictions $\hat{y}$ with a Gaussian PSF of experimentally individually determined radius and intensity. Thus

$$\begin{array}{c} L_{1}\left(G\right)=\lambda_{L1}E_{x,y}[{∥y⊗PSF-G\left(x\right)⊗PSF∥}_{1}]. \end{array}$$ (3)

To promote the sparsity in each frame produced by the generator, we add an additional L_1_ loss

$$\begin{array}{c} L_{1s}\left(x\right)=\lambda_{L1S}{∥G\left(x\right)∥}_{L1}. \end{array}$$ (4)

Thus the final loss-function is given by

$$\begin{array}{c} G=arg\;\min_{G}\max_{D}\lambda_{cGAN}\cdot L_{cGAN}\left(G,D\right)+\lambda_{L1}\cdot L_{1}\left(G\right)+\lambda_{L1s}\cdot L_{1s}\left(x\right). \end{array}$$ (5)

Additional hyper-parameters λ_*cGAN*_, λ_*L*1_ and λ_*L*1*s*_ are controlling the influence for each error term. The values for λ_*cGAN*_ = 3, λ_*L*1_ = 100, λ_*L*1*s*_ = 100 were chosen empirically, where we kept λ_*cGAN*_ = 0 for the first 1000 and then every third iterations to reduce any exploding gradients while training.

Compared to the Deep-STORM by Nehme et al. [31], the here presented GAN architecture is more flexible in terms of the cost-function. This is because it holds a data-specific regularizer which learns the properties of the unpredictable camera compression while training the generator.

Instead the discriminator tries to distinguish whether the results are coming from the generator or from the GT dataset. Hence the GAN should come up with a learned forward model which successfully includes all unknown effects, especially the compression artifact, to find the center of each fluorophore. This facilitates a parameter free optimization technique, well suited for the unknown black-box by the cellphone camera. Once trained, the localization is obtained by $\hat{y}=G\left(x\right)$.

#### 5.4.4 Generation of the training dataset

We used two different methods for a 50:50 mix of which generated the training dataset *x* to *y* to feed the modified version of the Pix2Pix GAN available at [53].

**Dataset from camera’s model simulation** We first created a data-stack of simulated STORM frames using the software ThunderSTORM. Parameters for data-generation were selected in accordance to experimental conditions. Emitters of varying densities (4-6 $\frac{\text{Particles}}{\mu m^{2}}$) were randomly distributed over the FOV. In a later step we estimated a camera model, based on the properties determined in Methods 5.3, to introduce noise into the data, before they were compressed by the H.264 video-codec in MATLAB [56]. The compression ratio was tuned, so that the compression artifacts looked similar to the one from the original acquisition (“Video-Quality”: 80 − 90%). The location of the H.264 integer blocks was not preserved when generating the dataset to avoid overfitting of the data due to the same grid-structure in each frame.

The compressed frames were decompressed and upsampled (in our studies we used a factor of 5×). The ThunderSTORM location positions were converted to the upsampled grid, rounded and a single pixel was set to the predicted brightness. This constituted the location maps.

Following this procedure gives only an estimated forward model of the unknown camera “black-box” and therefore cannot be expected to account for all properties of the data acquired by the cell-phone camera.

**Dataset from localized dSTORM data** Our second way to generate a dataset was by taking captured *cell*STORM time-series using the video-mode from real biological cells (labeled microtubules) under optimal conditions. After localizing the blinking events using ThunderSTORM in each decoded frame, we extract the detected emitters and generate a location map from this data.

To not only learn the forward model from the ThunderSTORM PSF-fitting algorithm, we also incorporated 50% data from the method described above. This makes our methods more robust to variations in the data. It also successfully accounted for variations in sample’s background as well as in the camera parameters.

#### 5.4.5 Testing

The GPU-based implementation of the localization image-generation algorithm allows fast multi-emitter processing and processes a video (e.g. 15 k frames, 128×128 pixels) at an upscaling-factor of 5 (to ensure sub-pixel super resolution) in about 5 Minutes (≈ 50 *fps*). On the other side the cellphone-based implementation can do 2-3 fps at 64×64 pixels input-frames, which can dramatically be increased by optimizing the code. Due to the convolutional-architecture of the PatchGAN-discriminator [57] it is possible to process data with frame-sizes different than the training dataset.

Our approach does not rely on any specific class of imaged objects, nor does it need any parameters other than a dataset which mimics the experimental data in the sense of acquisition parameters.

#### 5.4.6 Evaluation on the cellphone

During our study we tested three different implementations of Tensorflow’s mobile environment. The Tensorflow Mobile Library serves as a Java Native Interface and processes the network directly on the cellphones hardware using C-code. It is possible to improve execution speed by taking advantage of the device’s memory and load a batch-size of i.e. 10 images at once. Additionally we reduced the number of layers, the depth respectively, in the generator, so that there were only 4 encoder/decoder layers (256 → 128 → 64 → 32 → 32 → 64 → 128 → 256) left. This is possible, because the low-level features (e.g. detect a blinking event) occur only locally and do not interact globally in the FOV. This reduced the number of processable parameters and kept expensive memory transfer low to achieve frame-rates of up to 9 fps (frame-size of cropped region: 64×64 pixels up-sampled by a factor four).

The second library relied on the TF Lite framework which lacks necessary operands to fully work on the cellphone. A modified network, based on only available operands, did not show any improvement in computational time. It is worth mentioning, that the APP [42] runs on any Android cellphone, but computing time may vary significantly.

New cellphone hardware equipped with so-called Neuron Processing Units (NPUs) could potentially boost execution time significantly because a single convolution with an *n* × *n* filter-kernel can be carried out in one iteration, whereas standard CPUs need *n* ⋅ *n* CPU cycles. The Huawei HiAi framework (v. D150) allows to deploy pretrained NN graphs onto their NPU equipped devices (i.e. Huawei P20). Due to the lack of available operands (e.g. Absolute, Resize) it was not possible to prove the hypothesis of a speed improvement.

We would like to mention, that the overhead of loading the video-frames through the Java Interface can be dramatically reduced by proper hardware accelerated programming. Therefore the on-device processing of the acquired video-frames still serves as a proof of principle and is far away from being real-time compatible.

### 5.5 Cell culture

HeLa cells (ATCC CCL-2) cells were routinely cultured in ATCC-formulated Eagle’s Minimum Essential Medium (No. 30-2003) supplemented with 10.0% fetal bovine serum (No 30-2020) and 1% Penicillin-Streptomycin at 37.0°C in a humidified atmosphere with 5.0% CO2.

Fetal bovine serum to a final concentration of 10.
